# Newly Diagnosed Ulcerative Colitis in the Third Trimester: A Case Report

**DOI:** 10.7759/cureus.46004

**Published:** 2023-09-26

**Authors:** Arley K Rodriguez, Sean M Muir, Lindsay Tjiattas-Saleski

**Affiliations:** 1 Obstetrics and Gynecology, Edward Via College of Osteopathic Medicine, Spartanburg, USA; 2 Medicine, Edward Via College of Osteopathic Medicine, Spartanburg, USA; 3 Clinical Affairs, Edward Via College of Osteopathic Medicine - Carolinas Campus, Spartanburg, USA

**Keywords:** ulcerative colitis, c-section, preterm delivery, third trimester of pregnancy, nausea and vomiting in pregnancy

## Abstract

Ulcerative colitis is a chronic inflammatory bowel disease that results in continuous colon inflammation. It is uncommonly diagnosed during pregnancy, and if quiescent before pregnancy, becomes active very rarely. Current literature has found that amelioration of the disease can be common during the second and third trimesters. However, if ulcerative colitis is active, it has the potential to result in premature delivery and low birth weight. This case focuses on a rare presentation of primary ulcerative colitis diagnosed in a 22-year-old multiparous patient to highlight the importance of a comprehensive differential diagnosis in pregnant patients with seemingly benign symptomatology.

## Introduction

Ulcerative colitis (UC) is a chronic inflammatory disease that involves the mucosa of the rectum and colon, and it can extend proximally toward the cecum [1,2]. More than 750,000 Americans are affected by UC, with a higher prevalence reported among individuals with a white ethnic background in the US, Northern Europe, Canada, and Australia [1,2]. While there has been an increasing trend in incidence among men over the age of 45, women between the ages of 15-30 face the highest risk of developing the disease [1,2]. Although UC is most commonly recognized as an autoimmune disorder, its pathophysiology is still not well understood.

The most common symptoms of UC include bloody stool, diarrhea, urgency, and tenesmus. Other symptoms encompass mucus discharge, incontinence, abdominal cramping/discomfort/pain, and constipation [1-4]. Symptoms indicative of extensive colitis include classic inflammatory signs such as fatigue, fever, and weight loss. Common labs collected for UC include temperature, pulse, hemoglobin, C-reactive protein (CRP), erythrocyte sedimentation rate (ESR), and CBC. An elevated calprotectin level greater than 150-200 micrograms/g has been correlated with UC [1,3]. These labs, along with endoscopy, are necessary for diagnosing UC.

Classification of UC severity can be divided into mild, moderate-severe, and fulminant UC [1,3]. Mild UC consists of fewer than four stools/day, intermittent hematochezia, occasional urgency, normal hemoglobin, ESR < 30, and slightly elevated CRP [1,3]. Moderate-severe UC is characterized by greater than six stools a day with frequent urgency and hematochezia, hemoglobin levels below 75% of normal, ESR above 30, and elevated CRP [1,3]. Fulminant UC is defined by over 10 stools daily, continuous hematochezia and urgency, with ESR and CRP levels above 30 [1,3]. The Ulcerative Colitis Endoscopic Index of Severity (UCEIS) and Mayo subscore are both used to grade UC [1,5]. Although these classifications help determine the severity of the disease, they rarely dictate treatment options. Treatments follow a more systematic approach similar to other autoimmune diseases.

The goal of treatment is to limit UC flares and reduce hospitalizations/surgery, promote mucosal healing, and strive for steroid-free remission. Screening for osteoporosis, colorectal dysplasia and neoplasia, anxiety, and depression is recommended in patients with UC [1]. For mildly active UC, oral or rectal low-dose (2-2.4 g/day) 5-aminosalicylates (5-ASA), such as mesalamine or sulfasalazine, is recommended [5]. For mild to moderate cases, high-dose (4.8 g/day) oral 5-ASA can be considered if low-dose 5-ASA is insufficient. Mesalamine enemas have been reported to be more effective than oral 5-ASA for left-sided UC and may be beneficial [1,5]. If mesalamine or sulfasalazine proves ineffective, a diazo-bonded 5-ASA (balsalazide or olsalazine) may be beneficial. During UC flares, budesonide MMX 9 mg/day can be added [5]. For patients who have undergone >2 corticosteroid courses in the past two years or who are refractory to corticosteroids and/or 5-ASA, immunomodulatory options, such as thiopurines, vedolizumab, and tofacitinib, may be helpful [5].

A first disease attack of UC during pregnancy is uncommon and usually associated with adverse outcomes [6,7]. If a patient’s UC is in remission during the time of conception, it is likely to remain quiescent throughout the pregnancy, but if it is active, it has the potential to progressively worsen [6,7]. Active UC has been shown to be correlated with an increased incidence of stillbirth, preterm delivery, and postpartum complications for both mother and neonate [7,8]. Reports including many more than 100 pregnant patients with UC show normal, healthy offspring in 76% to 97%, congenital abnormalities in 0% to 3%, spontaneous abortions in 1% to 13%, and stillbirths in 0% to 3% [7,8]. The largest driver of adverse outcomes is an active disease, therefore active medical management should be pursued [7,8].

## Case presentation

A 22-year-old G3P1011 at 30w2d presents to an obstetric emergency room complaining of frequent water diarrhea with cramping and pubic pain. The patient states that this current episode has lasted two days but has had multiple episodes of nausea, vomiting, and diarrhea with this current pregnancy since 12 weeks. The patient has presented to the emergency department before with similar episodes that were resolved with intravenous (IV) fluids and oral Tylenol. The patient denies fever, chills, hematochezia, or unusual vaginal discharge. Current pregnancy complications include positive gonorrhea testing two months ago status post-treatment with Rocephin and azithromycin, as well as tetrahydrocannabinol (THC) use. The patient’s current medications included doxylamine, promethazine, pyridoxine, scopolamine, and a prenatal vitamin. The patient’s vital signs were within normal limits at this time. A cervical exam revealed 1 cm dilation, 60% effacement, and a fetal station of -3. Non-stress test (NST) was reactive with a baseline heart rate of 135 bpm and no decelerations or accelerations. Tocometry revealed some irregular uterine contractions. Urine drug screen positive for THC. Laboratory values were within normal limits, including complete blood count (CBC), complete metabolic profile (CMP), amylase, lipase, and urinalysis, except for minimally low potassium at 3.5 (normal 3.6-5.1 mEq/L) and elevated glucose of 184 (normal 70-99 mg/dL). Clostridium difficile polymerase chain reaction (PCR), Cryptosporidium antigen, Giardia antigen, and stool studies were negative. The patient was admitted to the hospital for overnight surveillance and given intravenous fluids, oral Tylenol, and oral potassium supplementation. Gastrology was consulted, reporting the patient was clear for discharge and felt unnecessary to proceed with further laboratory or imaging testing.

**Figure 1:**
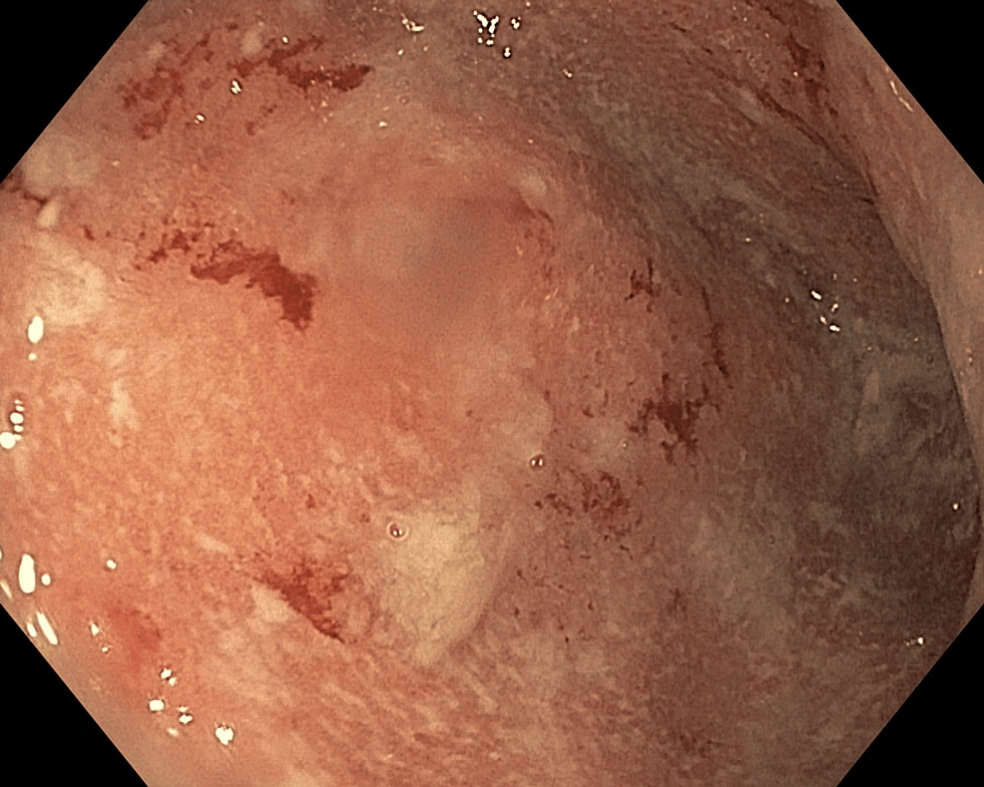
Flexible sigmoidoscopy revealing fulminant ulcerative colitis

The patient presented back to the obstetric emergency room four days later complaining of persistent abdominal pain and diarrhea. Upon her discharge, she was given anti-diarrheal and nausea medication but states that these have not helped. She further reports that over the past day, she noted blood intermixed with her stool. She reports that she had continually drinking water but was unable to keep fluids or fluid down. No extraintestinal manifestations were reported or observed. Vital signs were within normal limits. The cervical examination was unchanged from the last check. Laboratory testing did not show abnormalities. NST was reactive and tocometry revealed some irregular uterine contractions. Fetal biometric measurements showed normal estimated fetal weight, normal amniotic fluid volume, and no gross fetal anomalies identified, though 30 seconds of fetal breathing movement was not visualized during the exam. The patient was admitted at this time for symptomatic management, including in vitro fertilization (IVF) with K added, as well as continuing medication prescribed at a previous hospital visit. The gastrointestinal (GI) service was consulted to further investigate the patient’s symptoms. The patient underwent a flexible sigmoidoscopy revealing severe erythema edema friability and ulceration consistent with colitis in the sigmoid colon (Figure 1). The patient was started on 50 mg of IV hydrocortisone succinate every six hours.

The patient overnight and into the next morning stated that she was feeling better after starting the corticosteroid, but in the evening, NST showed minimal to moderate variability. A BPP was performed at the bedside with a score of 2/8, with the only points being given for normal amniotic fluid volume. The decision was to proceed with a primary low transverse urgent cesarean section with the risks, benefits, and alternatives discussed with the patient. The procedure had no complications with blood loss at 1006 mL. The patient produced a viable 2600-gram male infant with APGAR (appearance, pulse, grimace, activity, and respiration) of 9/9. The patient was stabilized in the hospital and discharged with prednisone 20 mg PO QD until able to follow up with GI to discuss a further treatment plan.

## Discussion

The investigation of the interplay between inflammatory bowel disease (IBD) and pregnancy has garnered interest due to the effects of pregnancy on microbiology and immunology. It is well-known that pregnancy influences the disease course of IBD. However, the physiological and immunological changes underlying these effects are not yet well understood. Recent studies have begun to explore the inflammatory and microbiome changes that occur during pregnancy, aiming to unravel why IBD symptoms might be ameliorated. Van Der Giessen et al. collected 46 fecal and serum samples from individuals with IBD (31 with Crohn's disease and 15 with UC) as well as 179 samples from healthy controls [6]. Samples were collected during pre-conception, the first, second, and third trimesters of pregnancy, and post-partum. The results demonstrated a suppression of the pro-inflammatory cytokine profile during pregnancy among patients with IBD [6]. This supports the idea that pregnancy exerts the most significant effect on UC in suppressing pro-inflammatory cytokines. Notably, differences in microbial diversity emerged during the second and third trimesters between patients with Crohn's disease, UC, and non-IBD pregnant patients [6]. Of similar interest, Miko et al. proposed that commensal bacteria are exchanged between the fetus and mother via the placenta, suggesting that diet could impact the fetal microbiome, influencing immune and metabolic diseases in offspring later in life [4]. These findings warrant further investigation and could be of much use when counseling UC patients having thoughts of conceiving.

It has been strongly suggested that patients conceive while their IBD is in remission, and equally important, they maintain remission during pregnancy and in the postpartum period [6,7]. This recommendation is due to active UC being shown to correlate with complications to the mother and fetus including risk of stillbirth, preterm delivery, congenital abnormalities, and intrauterine growth restriction [6,7]. First-line therapy for patients with acute severe UC includes hydrocortisone, which is considered safe in the second and third trimesters of pregnancy [7,8]. Corticosteroids are associated with a low risk of cleft palate [7,8]. Antibiotics are not indicated unless a specific pathogen is isolated [7,8]. Low molecular weight heparin is recommended for patients with UC after delivery due to their increased risk of venous thromboembolism [7,8]. There are a limited number of cases that have reported the use of salvage therapies for acute severe ulcerative colitis (ASUC) in pregnant patients [9]. Patients should be assessed for the need for salvage therapy if failure to respond to three days of intravenous (IV) hydrocortisone [9]. The drugs infliximab and cyclosporine are classically used for salvage therapy and are pregnancy class B drugs [9]. Anti-TNF therapies, such as infliximab, are not associated with risk to the pregnant mother or the fetus [9]. Colectomy is recommended in patients who fail to respond to salvage therapy within seven days [9]. Once successful induction is achieved, patients will require maintenance therapy, with the type being determined by their disease course [9]. Aminosalicylates (5-ASA) are commonly used drugs that are pregnancy class B, though there has been a risk of patent ductus arteriosus seen in third-trimester mice exposed to 5-ASA [9-11].

The aforementioned case presents a rare scenario of a multiparous pregnant woman diagnosed with new onset UC during pregnancy, whose symptomatology included common benign pregnancy complications of nausea, vomiting, and diarrhea. Many studies and clinicians support the notion that during pregnancy, there are calming effects on IBD symptoms and pathology, and the goal of this case report is to encourage clinicians to not let this belief limit working up benign symptoms such as in this case. Less than two days after the patient’s diagnostic flexible sigmoidoscopy revealed active fulminant UC, she started having non-reassuring fetal heart tracing and biophysical profile (BPP), which prompted an emergency C-section at 31 weeks. The patient had multiple clinical encounters where her symptoms were considered normal ailments of pregnancy and not worked up further. This delay in diagnosis and treatment could be correlated with the patient's preterm delivery, as active UC has been shown to increase its risk [6,7].

## Conclusions

Ulcerative colitis (UC) is a chronic inflammatory bowel disease that is uncommonly diagnosed during pregnancy, and if controlled before pregnancy, becomes active very rarely. This case presented a multiparous pregnant patient in her third trimester that presented with seemingly “common” GI complaints, ultimately revealing fulminant UC. Less than two days after the patient’s diagnostic flexible sigmoidoscopy, she started having non-reassuring fetal heart tracing and biophysical profile (BPP), which prompted an emergency C-section at 31 weeks. The goal of this case report is to raise awareness within the obstetrics community about the need for a comprehensive differential diagnosis when a patient presents with symptoms associated with pregnancy, such as nausea and vomiting, to avoid a late diagnosis of UC and other gastrointestinal (GI) conditions.
